# Identifying strategies and related principles supporting a co-design approach in an assistive device service delivery and research platform

**DOI:** 10.3389/fresc.2024.1364016

**Published:** 2024-07-23

**Authors:** Thuvaraha Jeyakumaran, Jordan Eggiman-Ketter, Abby Spadzinski, Dalton L. Wolfe

**Affiliations:** ^1^ Parkwood Institute Research, Lawson Health Research Institute, London, ON, Canada; ^2^ Faculty of Health Sciences, School of Health Studies, Western University, London, ON, Canada

**Keywords:** co-design, integrated knowledge translation (IKT), engagement, assistive device, rehabilitation

## Abstract

**Introduction:**

Possibilities Project Plus (PPPlus) is a free assistive device (AD) marketplace and research platform for persons with disabilities. The overall aim of PPPlus is to increase access to ADs through an integration of service, research and education. To maximize positive outcomes and reflect community needs a co-design approach informed by guiding principles of Integrated Knowledge Translation (IKT) was adopted, with examination of methods related to Experience Based Co-design. The integrated nature of PPPlus benefits from the use of specific engagement strategies that align with IKT principles to meet project objectives. The extent to which partnership and engagement strategies are specified in the rehabilitation research literature vary greatly and studies that provide information on specific strategies used to operationalize principles are limited. The objective of this manuscript is to provide a description of the co-design approach and the specific strategies that strive to achieve meaningful user engagement. By reflecting on these processes we also report on limitations and strategies for improvement.

**Methods:**

The co-design approach is highlighted through specific project activities including a representative governance structure, ongoing environmental scan and iterative Health Equity Impact Assessments (HEIA). The inherent engagement strategies that align with IKT and co-design principles are described.

**Discussion:**

The most impactful engagement strategies included early engagement of partners throughout all phases, ensuring project relevance across partners through alignment of objectives with complementary aims, using HEIAs to promote equitable outcomes from diverse stakeholders, the representative governance structure beyond individuals with disabilities and caregivers, and the use of experiences and stories to inform development.

**Next steps:**

This examination of specific strategies related to co-design focused on partnership engagement and informed targets for enhancement of the PPPlus initiative. These include being more intentional in developing a more rigorous process for evaluation that includes an assessment of strategies and their impact—especially as related to partner engagement. In addition, ongoing and enhanced efforts will focus on developing knowledge products that bring to life the most salient experience-based user stories emerging from the environmental scan with these being used to drive distinct co-creation events as well as serve other knowledge mobilization purposes (i.e., supporting policy change).

## Introduction

Persons with disabilities frequently depend on assistive devices (ADs) to enable their independence and enhance their overall well-being (1–3). It is estimated that 2.5 billion individuals world-wide are supported by at least one AD (4). However, there are numerous challenges to the procurement of these essential pieces of equipment such as limited income supports or inequities in service provision. In Canada, funding for devices is not federally legislated, and therefore is a provincial responsibility with many regional inequities and gaps (5). The Possibilities Project Plus (PPPlus) is a free AD marketplace and research platform intended to increase access to ADs (service) and provide evidence to establish the need and methods for enhanced support for persons with disability and their caregivers in accessing ADs in Canada (research). This ongoing initiative serves individuals in Ontario, with eventual plans to expand across Canada, and promote equitable health outcomes with an aim of supporting policy change through evidence.

PPPlus’ dual role as a service and research platform, serving as a vehicle for experiential learning, is enhanced through the integration of intentional engagement principles and underlying strategies designed to meet the needs of service-oriented, research and educational partners. The experiential learning approach involved incorporating students from a variety of disciplines throughout various aspects of PPPlus to gain their desired experience while building capacity of the platform.

To maximize positive outcomes for its intended users but also authentically reflect community needs, a co-design approach was deemed integral to the initiative's methodology, effectiveness and sustainability. This approach is further informed by Experience-based Co-design (EBCD) which has roots in Participatory Action Research (PAR) and emphasizes collaboration by involving the active engagement of staff, patients, trainees and other service users in co-designing services and care pathways (6–8). This strategy places significant value on the intentional gathering of user experiences and having stakeholders that are both service providers and end-users play an instrumental role in a shared approach to design, develop and implementation. The flexibility of the EBCD approach was a key advantage to ensure unbiased and specific priorities are identified through the sharing of experiences.

Several researchers have noted the many parallels (or areas of divergence) between various collaborative research approaches such as co-production (i.e., co-design), participatory research or integrated knowledge translation (IKT) (7, 9, 10). In particular, Nguyen et al. (9) demonstrated in an expert-based qualitative descriptive study comparing these approaches with IKT, that many have far more in common than not—with a common thread of the approaches being enabled through “true partnerships” (9). Recently, Gainforth et al. (11) have undergone a series of literature reviews and consensus-seeking activities to identify a core set of IKT guiding principles and related strategies to support quality research partnerships within the spinal cord injury research community (11–14). These guiding principles and strategies were intended to promote collaboration and foster engagement among researchers, policymakers, practitioners, and community stakeholders to allow for services and research findings that are relevant, applicable, and effectively implemented into practice (12). This context specific guidance co-developed with persons with lived experience aims to reduce tokenism in engagement strategies and empower individuals to contribute meaningfully.

These authors used dictionary definitions to signify partnership principles as “fundamental norms, rules, or values that represent what is desirable and positive for a person, group, organization, or community, and help it in determining the rightfulness or wrongfulness of its actions”, and strategies as “observable actions designed to achieve an outcome” (13). Although specific strategies are becoming more widely known and used, there is a lack of reporting on the specific engagement strategies associated with co-design or other collaborative research approaches, specifically in scientific articles (15, 16). Furthermore, the extent to which partnership and engagement strategies are described vary widely and studies that explicitly offer insights into the recommended strategies for operationalizing specific principles, particularly the critical aspect of linking principles and strategies are limited (13–15). The absence of such explicit guidance underscores a gap in current research. Reporting and evaluating specific strategies to engage various stakeholders will allow for better understanding on optimal methods for meaningful engagement for specific contexts, thereby enabling adoption of successful co-design and other research partnership approaches. The establishment of clear links between strategies and engagement principles will facilitate teams in enhancing their ability to enact these principles in a practical context (13).

In response to this research gap, the specific purpose of this paper is to describe the co-design approach implemented in PPPlus and the specific strategies designed and used to operationalize IKT principles. These acknowledge diversity and strive for representation and/or inclusivity as well as achieve meaningful research user engagement along with open and ongoing communication. In doing so, we hope to enhance our work toward an effective and responsive AD platform and research hub for persons with disability, with a specific emphasis on promoting equitable access to ADs.

## Methods—embedding co-design principles and associated strategies in research activities

Given PPPlus’ dual role as both a service and research platform that also serves as a vehicle for experiential learning opportunities, it was important to consider the intentional integration of IKT principles and associated strategies across the various activities of the initiative. Integrating strategies aligned with IKT principles aim to facilitate a cohesive co-design process that (1) engages all service users, (2) includes persons with lived experiences through leadership roles, and (3) enables research to be meaningfully translated into practice and service. The integration of these methodologies emphasizes the interconnectedness of patient-centered research with services, knowledge translation, and collaborative decision-making. These are intended to collectively contribute to the overarching goal of enhancing access to ADs. To illustrate this, we describe three key activities below that were seminal to the development of PPPlus and incorporated specific strategies aligned with IKT and co-design principles as well as identify future adaptations to these strategies. The three key activities include, (1) the formation of a representative governance structure that incorporates multidisciplinary perspectives from the project's inception, (2) ongoing environmental scans conducted across project phases and (3) iterative Health Equity Impact Assessments (HEIA). In this way, the emphasis of this section will address the stated gap i.e., describing the specific strategies we employed to facilitate engagement and co-design and their alignment with IKT principles.

### Representative governance structure

From initiation, PPPlus aimed to avoid tokenism, an overarching goal of the IKT principles, where various partners and groups have little say in research/program development by actively engaging and prioritizing perspectives from community organizations, individuals with lived experiences, and service providers (i.e., clinicians and others involved in AD access) to shape project practices. The co-design approach was incorporated in the project's commitment to a representative governance structure that included a diverse array of stakeholders as an intentional strategy involving the intersection of service, research and education. This strategy was embedded from the inception of the initiative which began with a founding partnership involving a community agency bringing together policy and service aims focused on persons with disabilities along with a research team embedded within a specialized rehabilitation centre and aligned with an academic institution. Importantly, the founding partners each brought their primary aims (aligned with their individual missions) to preliminary discussions in shaping the overall objectives of PPPlus such that they were highly relevant and enabled through the inherent strengths and resources of each partner. For example, the community organization has a strong track record in advocacy and enabling social justice efforts for persons with disabilities and has interest in the area of enhancing access to ADs for these individuals. The clinical and research partners shared this interest in ADs from a clinical and service delivery perspective as well as bringing strengths in implementation science and knowledge mobilization. The academic partner was interested in providing their students with experiential learning opportunities across these contexts ranging from involvements in clinical service delivery as well as research ranging from understanding impacts of ADs to informing social justice efforts related to access of ADs.

At the core of this structure is a Leadership team that spans individuals with lived experiences including persons with disabilities, caregivers, clinicians, policy makers, researchers, community partners, and students. This interdisciplinary team meets bi-weekly to deliberate on action items, fostering a comprehensive perspective that enables the IKT principle of shared decision-making.

Supplementing the core Leadership team are specialized working groups, some of which are led by students, focusing on crucial aspects such as marketing, environmental scanning, support agents, website development, risk / privacy, knowledge mobilization, research and evaluation. The student leaders are guided by members from the core Leadership team to direct specific working groups. The inaugural student leaders have been identified through separate projects where they had established their experience. Of note, the community partner brings expertise in policy change and social justice efforts as well as strengths in engagement strategies for persons with disabilities. Two team members from this group have significant expertise in policy/social justice efforts and also bring lived experience with disability which will be increasingly integral to the later stages of this work where we more actively translate research evidence into policy change efforts.

An important component of the strategy is the initiative's inclusion of students from a variety of undergraduate and graduate programs through experiential learning opportunities such that new ideas and diverse perspectives, as consistent with IKT principles, are injected into the initiative while building project capacity, productivity and sustainability as well as providing mentored experiential learning opportunities that facilitate capacity-building across many domains (i.e., clinical insight associated with ADs, service delivery, marketing, research skills, technology development).

Clinicians, including physiotherapists, occupational therapists and a clinical dietician, also play a crucial role within the project team. For example, a critical collaboration involved occupational therapists developing an AD database and determining relevant product specifications. This collaboration supported individuals to access the right products for their specific needs, facilitating enhanced transfers of equipment. Senior hospital leadership associated with the participating rehabilitation centre were also key stakeholders in the initiative. Although not participating in every core leadership meeting, a leadership representative (i.e., rehabilitation program coordinator) was identified and was able to facilitate engagement activities within the organization. In addition to providing a leadership perspective and facilitating clinician involvement, this has proved extremely helpful in leveraging other organizational consultations such as with Risk/Privacy and Communications Departments.

Another category of strategies that relates to the Governance Structure is reflected in the administrative and logistical supports, aligned with IKT principles of practical considerations, which were inherent in the way the leadership team and working groups operate. An online, inter-organizational, collaborative platform (Microsoft Teams) housed shared document organization, online meetings and discussion boards. In addition, meeting times and frequencies were determined relative to the ongoing team demands to maximize participation. This approach differed in its emphasis on transparency, as all members, regardless of their role and affiliation, were granted access to all materials. Consistent with IKT principles, this not only facilitated transparency but also fostered a culture of inclusivity and collaboration, enabling diverse perspectives to contribute to decision-making processes in an informed manner. Meetings involved having set agendas shared in advance with all involved individuals, recorded minutes and were structured to be as participatory as possible so as to facilitate shared decision-making. A key member of the team involved a staff Knowledge Mobilization Specialist, with experience in engagement of vulnerable populations, who could act as a facilitator to engage team members in discussions as well as in preparation of materials to promote knowledge sharing.

As the initiative matures, and as noted in the environmental scanning activity, additional partners are continually being identified and we are in development of an appropriate structure to ensure their involvement beyond active participation in the existing structures (i.e., Representative Core Leadership team and working groups). This is likely to take the form of an Advisory Team that meets regularly (e.g., quarterly) although an important strategy that governs continuing partnership engagement is that each partnership begins with an intentional series of consultations to identify mutual benefits, concerns and logistical issues along with a terms of reference that outlines expectations for working together. Another key strategy aligned with this is that partnership identification and engagement is iterative and aligned with the overall objectives of the initiative. For example, working group needs are communicated with the environmental scan team, who can then reach out to potential partners with *a priori*ty to explore emerging project needs.

### Environmental scan

In order to capture, understand and improve end-user experience and engagement as well as inform overall research objectives we conducted an ongoing environmental scan. The environmental scan involved a single semi-structured interview of approximately 90 min. Initial interviewees were identified through our founding partner who had undergone initial consultations with organizations in the disability community. From here a snowball method was employed where organizations identify other potential interviewees. An extraction template was used to generate a database including organizations’ objectives, who they served, and their experience with ADs. The environmental scan specifically focused on Ontario-based organizations and individuals involved in AD distribution, assessment, or procurement, involving online information capture, interviews with organizations and individuals, and an ongoing scoping review. Conducted over the past year, the scan aimed to evaluate the necessity for and provide insights into the development of the PPPlus platform. To date, the environmental scan has involved the capture of online information from 39 organizations, with 12 undergoing interviews to gain additional insights. Moreover, 15 individuals were interviewed individually or in focus groups.

Multiple organizations operate in this domain with similar objectives, however, in a fragmented manner. For example, many organizations from the environmental scan have reported the same issue of having ADs falling into disuse, ultimately ending up in landfills. The scan serves the purpose of identifying and recruiting potential future partners and by employing IKT and co-design principles move towards a coordinated solution, with the ultimate goal of effecting lasting change.

Multiple engagement strategies were used within the environmental scan of PPPlus which enriched the capability to gather information and insights from various perspectives (i.e., patient partners, community members, staff, students, etc.). One strategy used was the targeted recruitment of individuals and organizations through community and professional networks as well as online searches around ADs.

Throughout consultations, individuals would share experiences with ADs, perspectives on the current landscape of AD access and opinions on the need for a free AD platform, enabling the identification of gaps and needs, ensuring that the platform's design and services align with user requirements. Bate & Robert (8) describe stories and storytelling, as the foundation of EBCD, containing essential elements for a profound understanding of the present service and insights into what needs redesign for the future (8). As a result, another strategy was encouraging open sharing of stories and experiences with the interviewee, determining what is important for them to share and avoiding the use of stringent interview guides. By incorporating this narrative-focused strategy, we aimed to capture the individual experiences, recognizing the power of stories to inform and shape our understanding of the patient journey as well as other users or stakeholders related to this part of the healthcare system. Finally, to enable meaningful engagement with stakeholders, another IKT principle, we provide opportunities to explore formal partnerships that can be mutually beneficial. The shared exploration of partnerships allows individuals and organizations to carefully assess their involvement, determine the level of commitment that aligns with their goals, and evaluate whether the partnership is the right fit for them. This strategy facilitates the transition towards a “true partnership”. In facilitating a “true partnership”, connections between researchers and knowledge users are created throughout the entire research process to guarantee the benefits extend to all parties (9). While acknowledging the distinct contributions of both researchers and knowledge users, there is an equal appreciation for their unique perspectives (9). This marks a departure from situations where knowledge user engagement is confined to consultation or feedback at a specific moment, ensuring that knowledge users have the chance to actively participate in the decision-making process (9).

A fulsome qualitative analysis was not conducted. Rather, following each interview (i.e., which was also treated as an opportunity for facilitating ongoing engagement), the core team reviewed the meeting notes, engaging in collaborative discussions to identify key points of action and plan subsequent steps. The team would then initiate the implementation of these steps, taking into consideration the suggestions provided and assessing their feasibility. As an example, following a meeting with a community organization, a valuable suggestion emerged: to broaden our marketing focus beyond individuals with disabilities to include those who may know someone in need of a device. This suggestion came from an individual, representing an organization, sharing their experiences with trying to expand their organizational reach and the issues they were facing. While this was not formally measured, embracing this recommendation enhanced the scope of our marketing efforts, through identification of additional organizations and individuals.

### Health equity impact assessment

Another essential activity implemented in PPPlus is conducting equity assessments using the Ontario Ministry of Health and Long-Term Care's HEIA tool (17). This tool enables the integration of diverse perspectives with a specific focus on promoting equitable outcomes and mitigating any potential unintended negative impacts associated with the development of a platform like PPPlus.

To conduct the HEIA, a large group consensus meeting was organized with 23 participants attending both online and in person, including, students, clinicians, individuals with lived experience, care partners, hospital leadership, software programmers, and other stakeholders. The consensus process took place over the course of a full day. Diverse groups of participants engaged in small group-based discussions consisting of 3–5 individuals. The small breakout groups were structured to ensure there were diverse perspectives in each group (mix of clinicians, students, person with lived experience etc.) bringing together individuals with varied backgrounds and expertise. These discussions focused on addressing a range of equity-related questions. Guided by the HEIA template, the groups explored topics such as identifying affected populations, examining potential unintended negative impacts of the platform, proposing potential solutions and determining areas of priority.

The HEIA template itself played a crucial role by prompting a comprehensive examination of potential at-risk populations. This structured approach required participants to intentionally contemplate how the platform's implementation might impact various marginalized groups. The template guided participants to delve into detailed analyses with thoughtful considerations of social determinants specific to each demographic. By necessitating a thorough exploration of potential risks and impacts on diverse populations, the HEIA template not only encouraged nuanced discussions but provided a systematic assessment of the potential consequences associated with the platform's deployment. The HEIA provided a method for flexible and receptive tailoring of both the service and research as per the IKT principle, by creating risk mitigation strategies.

An engagement strategy operationalized here was the intentional design of small group discussions and subsequent report-back sessions to allow for every participant to have the opportunity to contribute their insights and lead conversations. This structure was pivotal to create a safe environment for engagement as reported in previous literature (15). In addition to fostering a platform for collective dialogue, the consensus process empowered stakeholders through the shared determination of priorities and solutions. This inclusive strategy enabled an open exchange of ideas and opinions, aligned with several IKT principles, resulting in the identification of key themes and priority areas that demand attention, especially concerning potential negative impacts. Actively involving stakeholders in decision-making not only amplifies diverse perspectives but also promotes power-sharing within the initiative, creating a collaborative environment where decisions are collectively driven and facilitating positive engagement (15).

Preliminary findings from this process identified potential adverse consequences associated with the development of PPPlus, including issues related to technological inequity, disparities in digital literacy, shifts in perceived government responsibilities, unnecessary or unsuitable equipment acquisitions, and challenges related to transportation barriers. Furthermore, key sub-populations identified included older adults, individuals with low socioeconomic status, rural residents, and minority communities. In response to the findings gathered thus far, a set of actions to mitigate inequities have been identified for priority impacts as deemed by the group. Among these, the creation of PPPlus Support agents stands out. The support agent roles are taken on by student volunteers supervised by members of the core Leadership team. These agents are accessible both in person and online, providing assistance in navigating the platform and addressing issues stemming from a lack of technology or digital literacy. The training process for the support agents consists of a set of standard operating procedures highlighting various scenarios and expected actions and continues to evolve as the platform grows. This proactive approach aims to mitigate potential challenges and enhance inclusivity within the PPPlus framework.

## Summary of strategies related to IKT principles and other collaborative research approaches

As noted earlier, there are similarities between various collaborative research approaches incorporating co-design, although separate literature reviews have shown a general lack of clarity or specificity in documenting specific strategies associated with the operationalization of over-arching principles such as effective ways to achieve representativeness, inclusivity and meaningful involvement of partners (7, 9, 13). Gainforth and colleagues have conducted a series of literature reviews and consensus activities to identify a set of guiding principles associated with one of these collaborative approaches (IKT) (12–14). Hoekstra et al. (18) extended this work by proposing a system—based on the acronym “RECIPE”—that enables characterization of specific strategies in alignment with these guiding IKT principles (18). Table 1 employs an adaptation of this approach to summarize the embedded strategies within our own work in PPPlus involving an integration of service, research and educational activities to enhance access to ADs for persons with disabilities. This table provides an explicit summary of the engagement and co-design strategies we employed, structured to reflect their alignment with Hoeksta et al.'s IKT principles (12, 18). Of note, we also examined methods of EBCD with a view to further enhancements.

**Table 1:** Specific strategies employed in PPPlus linked to the IKT guiding principles and “recipe” concept (11–14).

“Recipe” categories	Strategies
• Related IKT principles	
**R**esources and time • Logistics & practical considerations^a^	• Meetings and engagement activities structures around members schedule's • Multi-faceted approach to provide in-kind or paid support for members • Development of student “agent” team to provide support as needed
**E**ngagement strategies (fostering collaborative/communication processes) • Ensure all voices are heard^a^ • Empower community members • Foster shared decision-making and co-ownership/co-production^a^	• Encourage open sharing of stories and experiences rather than structured interview guides with participants (or partners) • Use of targeted strategy to identify and engage organizations and individuals in AD space ⚬ Enabled by growing community network through partnerships ⚬ Further supported by online searching/environmental scan activities • Shared creation of solutions and identification of priorities • Emphasis on facilitative leadership style across teams
**C**ommunication activities & methods • Open and ongoing communication^a^	• Most activities are supported by an online collaborative platform (Microsoft Teams) supporting shared document organization, online meetings and various communications functions (e.g., archived chats and discussion forums)
**I**nitiatives for collaborative activities • Ongoing monitoring and evaluation of collaborative research activities^a^ • Facilitate ongoing knowledge mobilization	• Consensus meetings typically include small groups to facilitate comprehensive dialogue and sharing from all perspectives (e.g., HEIA process employed consensus methods involving structured nominal group and break-out groups) • Use of Re-Aim Framework (19) including a process component for ongoing monitoring and evaluation (planned) • Meetings have set agendas, recorded minutes and structured to be as participatory as possible
**P**artnership representation/relationships • Facilitate relationships based on respect, trust and credibility (avoid tokenism)^a^ • Partners involved in any & all phases of initiative (including early) • Ensure research is relevant and involvement of members is meaningful^a^ • Co-development of norms, rules and expectations	• Governance structure is representative and organized to facilitate collaboration and member involvement ⚬ Identification and supported involvement of “Game-Changers” i.e., innovators that are flexible and promote new ways to work and collaborate e.g., Policy Specialists who also bring lived experience, Senior Administrator leveraging organizational resources, Implementation Scientist, Knowledge Mobilization Specialist, IT Specialist cross-trained in Research Coordination/Project Management
	• Comprehensive strategy (including environmental scan) to identify partners and recruit individuals from different disciplines, backgrounds and sectors ⚬ Marketing strategy (including through community partners) targets research participants, some of which may become partners • Core project team and working groups includes members from policy sector, persons with disabilities, researchers, clinicians, students, community organizations • 29 Students support this initiative from various disciplines (Health Sciences, Occupational Therapy, Medical Sciences, Global Health, Business and Computer Science) • Consult with partners and research users collaboratively to agree upon level of commitment and engagement • Involvement of a knowledge mobilization specialist to facilitate collaborative processes
**E**ducation and training	• Corporate onboarding available for all members (staff, volunteers, persons with disabilities, students) • Content specific resources created (e.g., AD training package)
**O**ther • Partners address ethical challenges related to collaborative research activities^a^	• Iterative HEIA processes as consensus exercises to identify ethical challenges and sources/solutions related to inequity • Corporate risk and privacy consultation • Routine exploration of issues related to ethics, inequitable access to ADs and logistical challenges in environmental scan and partnership discussions

^a^ Related IKT principle (12, 18).

## Discussion

Co-design methods have become increasingly employed within the design and implementation of research or quality improvement initiatives. These are often a key component of collaborative research approaches that involve meaningful involvement of partners, and although structured processes are often involved, there is a general shortcoming in the literature in reporting the specific methods associated with co-design and partnership engagement or involvement (13–15). In the present manuscript, we were informed by Gainforth and colleagues work that identified a set of guiding IKT principles (12, 18) to more intentionally describe the specific strategies that enabled an integrated research, service and education initiative designed to enhance access to ADs for persons with disabilities. Specifically, we used Hoekstra et al's (18) “RECIPE” system to identify specific strategies that were used in this initiative to enable the co-design approach with a focus on partnership engagement and ongoing collaboration (see Table 1). In examining literature addressing principles and underlying strategies related to collaborative research (including co-design) it is evident there is significant overlapping of concepts and nomenclature. Also, although there is little detailed information on how specific strategies may operationalize principles, this was evident in our own work such that a given strategy may relate to multiple principles. This suggests that one should consider a combination of strategies to bring key principles to life. We also outlined three key research activities (representative governance structure, environmental scan, equity assessment) for this initiative and described how the specific strategies for co-design and partner engagement were implemented within these.

In striving to create and continually improve a service that also incorporated research and experiential learning opportunities for students, we sought to integrate co-design as a fundamental practice, yet also noted the need to establish processes that enlarged the focus beyond gathering perspectives and data limited to only the patient experience (20). Rather, this was enhanced to address all end-users and other stakeholders. Moreover, patients and other stakeholders should not be viewed as mere sources of information but as authentic partners in the process, recognizing their genuine contributions and collaboration (20).

Previous literature highlights the most frequently reported principles and strategies, but within the context of PPPlus, certain strategies stood out as particularly impactful, leading to tangible changes resulting from engagement and informing co-design. This included involving partners in all phases, especially in the early stages, facilitating comprehensive integration where partners could actively contribute to development well before the launch. By doing so, partners were involved in decisions from the project's inception rather than as an ‘afterthought’ or restricted to consultant roles as often described (9, 20). The partnership with the community organization with experience supporting individuals with disabilities as well as bringing expertise in policy change will be instrumental to the project as this aligns with the ultimate objective of the initiative. This partnership allowed for a synergistic blend of expertise, ensuring that the unique needs and perspectives of the target population were consistently considered throughout development and maintained the commitment to eventually bringing about policy change through social justice efforts. This approach not only prioritized the voices of service and knowledge users but also reinforced the project's broader mission of creating lasting positive change within the community.

Previous articles surrounding engagement approaches that we have encountered have not covered a structured strategy to address ethical concerns or promote equitable outcomes (18). The HEIA used in PPPlus provided a chance for various stakeholders with diverse backgrounds and expertise to intentionally consider how inequities may be perpetuated through this work and actively generate solutions to mitigate this. By continuing to conduct HEIAs and engage stakeholders in this comprehensive process, intentional equity considerations will continue to be at the forefront of the platform's design and promote the representativeness of the involved stakeholders.

This approach empowers stakeholders to contribute their insights and expertise, promoting equity, and enabling the project team to address potential challenges proactively. A fundamental aspect of this framework is that it provides a logical pathway from identifying potential ethical concerns regarding inequities to considering solutions that are grounded in social determinants and perspectives related to marginalized groups. However, recognizing the potential for improvement, future iterations could benefit from more intentional involvement of representatives from specific marginalized groups to further enhance the depth and relevance of the analysis. By continually refining these processes, we aim to maintain our commitment to inclusivity, equity, and the consideration of social determinants in our ongoing efforts to address the needs of diverse populations.

Another important strategy was surrounding the representative governance structure. Often it is individuals with disabilities and their caregivers collaborating in rehabilitation research, especially concerning service delivery where other stakeholders are underrepresented (15). However, other stakeholder groups provided the potential to contribute significantly by leveraging their distinct perspectives and skills. PPPlus recognized the potential of a diverse engagement strategy, partnering with a community organization to expand reach and also engaging important stakeholders from front line clinicians, senior leadership, policy sector and more—boosting sustainability and feasibility. Importantly, by integrating senior leadership from our hospital partner as an active role on the service delivery side, we were able to secure a designated leadership contact. This individual played a crucial role in navigating alignment with the rehabilitation center's goals, facilitating a more cohesive and strategic approach to service delivery. Senior leadership, as well as participation from an operational manager, enabled discussions with the organizational Risk and Privacy department to establish safe practices and minimize liabilities.

Finally, by adapting the traditional environmental scan and information gathering efforts to focus on sharing experiences and stories PPPlus is able to capture a nuanced understanding of one's experiences. In this process, narratives have proven to be catalysts for meaningful discussions, guiding development processes which is an integral part of an EBCD approach, although future efforts may extend this aspect in line with a more rigorous approach to storytelling as part of a more formal co-design event (6, 21). The environmental scan also played a crucial role in enabling the recruitment of specific partners, the identification of strategic approaches to connect with other collaborators and the utilization of various networks to broaden outreach.

In addition to the strategies noted above felt to be most impactful for the context of PPPlus, it is worth noting how these compare with findings from other researchers. In their scoping review, Hoekstra et al. (13) noted that the most frequently reported strategy was having structured meetings (whether face-face, phone or conference calls). We identified this to be useful as well. Heaton et al. (22) reported on a large collaborative initiative conducted by the National Institute for Health Research across England which identified nine core mechanisms characterizing “closer collaboration”. In addition to the importance of initiatives being driven by local end-users they noted that having “game-changers” and “facilitative leaders” as part of a small strategic core team are integral to success. “Game-changers” are innovative and find new ways to do work and “facilitative leaders” have real and perceived credibility combined with enthusiasm and perseverance along with a style that encourages partnership involvement. The ability of the team to bring together various partners that represent the requisite “creative assets” is another mechanism. In the PPPlus context, these mechanisms have been instrumental to success as we have brought together innovators (“game-changers”) and several facilitative leaders that comprise a rich blend of expertise and knowledge across domains related to policy, implementation science, knowledge mobilization, information technology, administration, experiential learning, clinical subject matter expertise (i.e., ADs) and lived experience.

Overall, the nature of PPPlus as both a service and research platform along with a focus on experiential learning presents a unique need to incorporate research, service and education (i.e., student) engagement strategies. The IKT framework was designed with research partnerships in mind, aligning with other collaborative research approaches (e.g., PAR). On the other hand, EBCD directs its emphasis towards quality improvement and/or implementation related to service. Our overarching objective within PPPlus is on an integration of service and research while providing an experiential education component. Therefore, some principles/strategies are likely to be appropriate, but there may be some oversights or specific strategies that may also be more helpful with these various contexts. This complexity may prompt us to explore alternative considerations and strategies that can enhance our multifaceted approach where these approaches may not fully serve one context or another. A significant challenge for our own work lies in this complexity and the need for a broad representation and diverse skill sets and perspectives to be included in the various engagement and co-design strategies. For those planning collaborative approaches involving co-design, an important consideration is to think about the overall context and objectives of the initiative and consider those strategies that seem most likely to link with the desired principles but noting that it all starts with meaningful engagement and a shared vision from the outset.

## Limitations

The co-design approach implemented by PPPlus, while informed by various engagement strategies, is not without its limitations. Three key challenges are evident, namely the time-consuming nature of the process, difficulties in fully representing all relevant stakeholders, and balancing the engagement strategies of service and research along with involving an experiential learning component.

The co-design approach, with its emphasis on engaging multiple stakeholders and incorporating iterative feedback loops, is inherently time-consuming. The thoroughness of the process demands a significant investment of time and resources. The challenge lies in balancing the need for a comprehensive co-design with the urgency of addressing the immediate needs of individuals with disabilities. The dedication of organizational time and resources to this process is substantial, and there may be inherent tensions between the thoroughness of co-design and the pressing demand for timely solutions. PPPlus aimed to address this by leveraging an experiential learning model where students are able to gain experience they are interested in, while supporting the PPPlus through specific working group tasks.

In addition, despite efforts to engage a diverse range of stakeholders, including individuals with lived experience of disability, caregivers, clinicians, policy makers, researchers, community partners, and students, the challenge of fully representing all stakeholders persists. Hard-to-reach groups, often marginalized or facing barriers to participation, may not be adequately represented in the co-design process. Persons with disability often face significant barriers to participation with challenges often associated with transportation, limited energy and having to manage secondary health complications. The engagement process undertaken aimed to be as accessible as possible following the schedule of our partners, offering online or in person engagement sessions/meetings and communicating with them in their preferred way. This is accomplished through intentional conversations with all stakeholders. It's essential to acknowledge that the inability to include all groups thoroughly due to various constraints does not render the co-design process irrelevant, nor should it be a reason to delay action. The co-design process should be viewed as iterative and open to improvement over time. Implementing agile principles can help mitigate time constraints by allowing the project team to respond to changing needs, reprioritize tasks, and iteratively build upon previous work in a more flexible manner. While it may not be possible to reach out to every potential stakeholder or impacted group in the initial stages, ongoing efforts to expand representation should be an integral part of the project. Feedback from the initial stages can inform strategies for reaching and including hard-to-reach groups in subsequent iterations.

Finally, PPPlus functioning as both service delivery and research requires use of engagement strategies related to both domains, in addition to offering experiential learning opportunities. Given this, engagement approaches may not have been entirely applicable to each aim resulting in a potential impact of less than complete adherence to respective processes that were intended to target each area. It will be crucial to continue evaluating and determining what works best across this integrated context.

## Conclusions/next steps

The co-design approach adopted by PPPlus is characterized by engagement and partnership strategies aimed to align with principles consistent with IKT with additional linkages to methods associated with EBCD. These strategies are highlighted through three key activities, a representative governance structure, ongoing environmental scan, and iterative HEIAs to achieve overarching IKT principles of acknowledging diversity, striving for representation and/or inclusivity, creating meaningful research and service user engagement and fostering ongoing communication.

In response to the current discourse on engagement within the rehabilitation setting, there is a growing need for heightened transparency regarding specific strategies that operationalize these fundamental principles. The present manuscript attempted to address this need by detailing specific engagement strategies and demonstrating their relationship to specified principles that enable co-design associated with an integrated service, research and educational initiative. However, there is still a need for guidance regarding the specifics of reporting and the details to best operationalize principles. The field would benefit from development of a standard reporting framework, although we believe the “RECIPE” approach suggested by Hoekstra et al. (18) is a useful step in that direction.

Going forward, we plan to intentionally evaluate the effectiveness of the engagement strategies by employing process and impact evaluations informed by the RE-AIM Framework (19) and also focused on the perspective of our partners and adapting strategies accordingly. Importantly, we underscore the significance of comprehensive reporting on limitations, recognizing that this transparency is essential for adaptive strategies and future planning of other studies and projects. By acknowledging limitations, PPPlus aims to foster an environment conducive to continual improvement and innovation in the pursuit of effective engagement and partnership in the rehabilitation context.

Drawing from EBCD frameworks more, a shift towards more intentional narrative driven co-design events to maximize impact and broader involvement is another approach that we intend to conduct. The significance of user stories and experiences in the EBCD process can be captured during workshops or individual interviews and disseminated to those engaged as well as to future stakeholders which will further establish needs and ensure seamless continuation of a transparent co-design process. This will likely include exploring diverse knowledge dissemination strategies involving stories to enhance the co-creation of events but also serve as the foundation for the policy change.

## Data Availability

The original contributions presented in the study are included in the article/Supplementary Material, further inquiries can be directed to the corresponding author.
